# Optimization on biodistribution and antitumor activity of tripterine using polymeric nanoparticles through RES saturation

**DOI:** 10.1080/10717544.2017.1410260

**Published:** 2017-12-01

**Authors:** Juntao Yin, Peiqing Wang, Yuyun Yin, Yantao Hou, Xiaoyong Song

**Affiliations:** a Department of Pharmaceutics, Huaihe Hospital Affiliated to Henan University, Kaifeng, PR China;; b Henan Provincial Institute of Food and Drug Control, Zhengzhou, PR China;; c Henan Vocational College of Applied Technology, Dongjing Avenue, Kaifeng, PR China

**Keywords:** Tripterine, nanoparticles, pharmacokinetics, biodistribution, RES saturation, antitumor

## Abstract

Systemic delivery of tripterine (TPR) is challenged by its insoluble property and unsuitable pharmacokinetics. This work aimed to develop polymeric nanoparticles (NPs) combined with the reticuloendothelial system (RES) saturation to improve the *in vivo* distribution and antitumor activity of TPR. TPR-loaded nanoparticles (TPR-NPs) were prepared by the low-energy emulsification/evaporation method and characterized with particle size, entrapment efficiency, and morphology. The resulting TPR-NPs were 75 nm around in particle size and displayed a sustained drug release in pH 7.4 medium. Pharmacokinetic studies revealed that TPR-NPs had the advantage in bettering the pharmacokinetic properties of TPR over the solution formulation. However, the ameliorative effect on pharmacokinetics was more significant in the case of RES saturation (i.e. preinjection of blank NPs). Preinjection of blank NPs followed by injection of TPR-NPs resulted in higher distribution of TPR into the tumor due to reduced sequestration of TPR-NPs by RES. In tumor-bearing mice (prostatic cancer model), TPR-NPs treatment with RES saturation exhibited a superior antitumor efficacy to free TPR and TPR-NPs alone. It can be concluded that formulating TPR into polymeric NPs in combination with RES saturation is an effective means to address the systemic delivery of TPR.

## 1. Introduction

In the last decades, phytomedicines have gradually stepped into the development framework of new medications. However, most entities isolated from medicinal plants are provided with poorly water-solubility and/or biomembrane permeability (Williams et al., 2013), which greatly limit their clinical successes. Tripterine (TPR), also known as celastrol, is an inhibitor of proteasome extracted from the Chinese herb *Tripterygium wilfordii*, which demonstrates multiple antitumor activities, such as breast cancer (Mi et al., 2014), lung cancer (Wang et al., 2015), and prostate cancer (Guo et al., 2016). Unfortunately, unfavorable physicochemical and pharmacokinetic properties such as low solubility, poor bioavailability, and systemic toxicity compromise its therapeutic benefits. Although some nanoparticle-based formulations have been employed for the bioavailability enhancement of TPR (Zhang et al., 2014b; Li et al., 2015), the plasma drug levels remain to be inadequate for chemotherapy. Intravenous administration will become an inevitable option if it exerts the antitumor effect of TPR. However, poor solubility and pharmacokinetics are the limited factors that have to be overcome.

Nano-drug delivery systems have been established able to potentiate drugs and address the formulation challenges of ‘problem’ pharmaceuticals (Mattheolabakis et al., 2012). For the recent years, a variety of nanocarriers have been proposed for systemic delivery of insoluble drugs, including liposomes (Allen & Cullis, 2013), micro/nanoemulsions (Gupta, 2011), micelles (Lu & Park, 2013), lipid-based nanoparticles (Grinberg et al., 2014), and polymeric nanoparticles (Xu et al., 2013). Among these, polymeric nanoparticles are widely concerned by pharmaceutical practitioners due to good biocompatibility and physiochemical stability (Zhao et al., 2016). However, plain nanoparticles have low-distribution specificity after injection and are easily captured by the mononuclear phagocyte system (MPS) (Aoyama et al., 2016; Li et al., 2016), which inherently compromise the *in vivo* delivery performance. Ligand decoration can improve the pharmacokinetic properties of nanoparticles (NPs) to some extent and thus enhance the therapeutic index of payload, though the surface engineering of NPs involves sophisticated chemical processes and high costs (Bi et al., 2016). Therefore, more practicable approaches are still desired for systemic drug delivery, especially for those drugs with poor solubility and oral absorption.

In this study, TPR-loaded NPs (TPR-NPs) were developed and a RES saturation strategy was explored for optimizing the pharmacokinetic and antitumor efficacy of TPR. TPR-NPs were prepared through a low-energy emulsification/evaporation method and characterized with particle size, entrapment efficiency, *in vitro* release and cytotoxicity. Pharmacokinetics and biodistribution of free TPR, TPR-NPs, and NPs/TPR-NPs (preinjection of blank NPs followed by injection of TPR-NPs) were evaluated in rats and tumor-bearing mice, respectively. The antitumor effects of free TPR, TPR-NPs, and NPs/TPR-NPs were investigated in prostate cancer xenograft mice.

## 2. Materials and methods

### 2.1 Materials

Tripterine was obtained from Baoji Herbest Bio-Tech (Baoji, China). Polycaprolactone (PCL, average *M*_w_ ∼14,000) was purchased from Sigma-Aldrich (Shanghai, China). Polysorbate 80 (Tween 80), ethyl acetate and 3-(4,5-dimethyl-thiazol-2-yl)-2,5-diphenyl-tetrazolium bromide (MTT) were provided by Sinopharm Chemical Reagent (Shanghai, China). RPMI 1640 medium and penicillin-streptomycin were purchased from Gibco® BRL (Gaithersberg, MD). HPLC-grade methanol was from Merck (Darmstadt, Germany). Deionized water was produced by a Milli-Q purifier (Millipore, MA). All other chemicals were of analytical grade and used as received.

### 2.2 Preparation of TPR-NPs

TPR-NPs were prepared through the low-energy emulsification/evaporation method based on pseudoternary phase diagram (Fornaguera et al., 2015). Briefly, the aqueous phase (pH 7.4 PBS) was dropwise added into the organic phase consisting of ethanol/ethyl acetate (20/80), 5% PCL and Tween 80. The region of O/W nanoemulsions occurring was visually investigated through the pseudoternary diagram of water/surfactant/oil. Different colloidal emulsions were discriminated by their appearance whether being transparent, translucent or slightly opaque, of which the sample with bluish shine was designated as nanoemulsions. To further confirm the formation of nanoemulsions, their particle size was simultaneously monitored by dynamic light scattering as described below. Next, the optimal nanoemulsion formulation was used to load TPR by adding TPR into the organic phase. TPR-loaded nanoemulsions were evaporated under reduced pressure to remove the organic solvents. Finally, TPR-NPs with ultrafine size were obtained by dialyzing them against deionized water to remove redundant surfactants. The effect of TPR amount used in the formulation on particle size and drug loading of TPR-NPs was screened.

### 2.3 Characterization of TPR-NPs

The particle size and ζ potential of TPR-NPs were measured with Zetasizer Nano ZS (Malvern, Worcestershire, UK) at 25 °C based on dynamic light scattering (DLS). The sample was diluted to 100 times with deionized water and subjected to laser diffraction or Doppler velocimetry for particle size and ζ potential output. The data were processed with the build-in software.

The morphology of TPR-NPs was inspected by transmission electron microscopy (TEM, Philips Tecnai 10, Amsterdam, The Netherlands). TPR-NPs were dropped on a carbon-coated copper grid and fixated by evaporating the dispersate under strong light. After sputter coating with gold, the surface of nanoparticles (NPs) was scanned with TEM, and TEM micrographs were taken at an acceleration voltage of 100 kV.

### 2.4 Determination of entrapment efficiency

The entrapment efficiency (*EE*) of TPR-NPs was determined by size-exclusion chromatography using a Sephadex G50 column (Zhang et al., 2014a). Free or unentrapped TPR was separated from the nanosuspensions against a 30 cm-long column with 0.5 mL loading volume. The anterior portion of outflow with an apparent opalescence, standing for pure TPR-NPs, was collected and analyzed for TPR concentration. *EE* was defined as the ratio of entrapped TPR (*W*_ent_) to total TPR (*W*_tot_), namely *EE* (%) = *W*_ent_/*W*_tot_ × 100%. Determination was performed in triplicate. TPR in the samples was quantified by the Dionex Ultimate 3000 HPLC system (Thermo Scientific, Waltham, MA). Samples were eluted on an Acclaim^TM^ C18 column (5 μm, 4.6 mm × 250 mm) with 20 μL of injection volume and detected at 425 nm. The mobile phase comprised 95% methanol and 5% water with 0.25% phosphoric acid pumped at 1.0 mL/min.

### 2.5 *In vitro* release study

Tripterine release from TPR-NPs was tested at 37 °C by reverse bulk equilibrium dialysis method (Ye et al., 2015). A number of dialysis bags containing 1 mL of blank medium were put into 250 mL of release medium (pH 7.4 PBS with 0.5% Tween 80 as solubilizer). Afterwards, 2.5 mL of TPR-NPs were added into the bulk phase outside the dialysis bags. After equilibrium under agitation, the dialysis bags were withdrawn at predetermined intervals. TPR concentration in the dialyzates was determined by HPLC as described above, and the accumulative release percentage was calculated.

### 2.6 *In vitro* cytotoxicity assay

The *in vitro* cytotoxicity of TPR-NPs was evaluated on LNCaP cells, a prostatic cancer cell line. LNCaP cells were from Chinese Academy of Sciences Cell Bank (Shanghai, China) and cultured in RPMI 1640 medium according to the reported procedure (Chen et al., 2016). LNCaP cells (passage 8–12) were seeded in 96-well plates at a density of 5 × 10^3^ cells/well and cultured for 48 h. Then, free TPR, blank NPs, TPR-NPs, or NPs/TPR-NPs diluted with culture medium in advance were added into the cells. In the case of NPs/TPR-NPs, NPs were first added to the cells, and 0.5 h after that, TPR-NPs were added. The cells were treated for 24 h with different TPR level (0.5, 1.0, and 2.0 μg/mL) at 37 °C. Finally, the cell viability was detected by MTT assay (Ji et al., 2016).

### 2.7 *In vivo* pharmacokinetics and biodistribution studies

Male SD rats weighing 220 ± 20 g were randomly divided into three groups (*n* = 5), i.e. TPR solution, TPR-NPs and NPs/TPR-NPs (injection of NPs followed by TPR-NPs). The rats were anesthetized using chloral hydrate and then cannulated in the site of jugular vein. TPR solution (dissolved in a cosolvent comprising propanediol/ethanol/water =5/3/2, v/v) and TPR-NPs were intravenously administered to the first two groups of rats via the jugular vein at a dose of 2.5 mg/kg equivalent to TPR. The third group of rats were previously injected with blank NPs and then given TPR-NPs after 30 min. At predetermined time points, aliquots of blood (∼0.25 mL) were collected into the heparinized tubes via the jugular vein and then centrifuged at 5000 rpm for 5 min to collect the plasma. The procedure of plasma TPR extraction followed the reported method (Li et al., 2015). TPR extracted from the plasma was quantified using a UPLC–QTOF/MS (Xevo G2 QTOF, Waters, Milford, MA). The instrument parameters and configuration were the same as the literature (Li et al., 2015).

The tumor-bearing BALB/c mice were used to study the biodistribution of free TPR and TPR-NPs. The construction of prostatic tumor model referred to the publication (Chen et al., 2016). Briefly, male BALB/c mice were inoculated subcutaneously with LNCaP cells at the right flank. When the tumor grew to 200 mm^3^ (*V* =* L* ×* W*^2^/2) around, the mice were randomly divided into three groups as abovementioned. The first two groups of mice were directly injected with TPR solution or TPR-NPs through the tail vein at a dose of 1 mg/kg. The third group mice were previously injected with blank NPs and then injected with TPR-NPs after 30 min. At 1 and 8 h after administration, the mice were sacrificed by cervical dislocation. Tumor and vital organs including heart, liver, spleen, lung, and kidney were excised from the mice. After cleaning and weighing, the tissues were homogenized with normal saline (2 mL/g of tissue). TPR was extracted from the homogenates with five aliquots of methanol and quantified by UPLC-QTOF/MS.

### 2.8 *In vivo* antitumor effect evaluation

The antitumor efficacy of TPR-NPs was evaluated on BALB/c mice (20 g ± 2 g) with xenograft of LNCaP cells in the right flank (Chen et al., 2016). The tumor-bearing mice were separately injected with physiological saline, TPR solution, TPR-NPs, and NPs/TPR-NPs (∼75 nm in particle size) through the tail vein every other day at a dose of 1 mg/kg. The tumor volume and body weight of each mouse were recorded every 2 d. The treatment lasted for 14 d. Finally, all mice were sacrificed and photographed, and the tumors were taken out and weighted. The histomorphology of tumor masses was examined by H.E. staining.

## 3. Results and discussion

### 3.1 Preparation and characterization of TPR-NPs

Nanoemulsion-template technique can produce smaller NPs in comparison with other preparative processes (Margulis-Goshen et al., 2010; Ishak et al., 2017), such as ultrasonic dispersion and high-pressure homogenization. In this study, the low-energy emulsification-evaporation method based on nanoemulsions was explored to prepare TPR-NPs. We utilized a pseudoternary diagram to find the region of nanoemulsions formation (Figure S1). Blank nanoemulsions (O/W) approximately took place on the water phase (W) ratio ranging from 53% to 82%, oil phase (O) ratio ranging from 1% to 20%, and surfactant (S) ratio ranging from 12% to 38% through visual observation. For intravenous purpose, more surfactants resided in the formulation are harmful to the body, but less use of surfactant will lead to larger size of NPs. In order to obtain a suitable particle size, it had to compromise the ratio to 25% for production of nanoemulsions. Afterward, the water and oil phase ratios were optimized by the hydrodynamic size (by DLS) and appearance (by eyesight). The formulation composition that could result in smaller particle size and transparent appearance was determined, as indicated by the red dot in Figure S1.

The preferred nanoemulsions composition (25% S, 61% W, and 14% O) was subsequently used to load TPR for final preparation of TPR-NPs. In addition to dissolving TPR into the oil phase, the preparation procedure of TPR-loaded nanoemulsions was the same as blank nanoemulsions. The prepared TPR-loaded nanoemulsions were then subjected to evaporation followed by dialysis to remove the organic solvents and redundant surfactant. After that, TPR-NPs were obtained. The effect of drug/PCL ratio on particle size and *EE* of TPR-NPs is shown in Figure S1. The ratio of drug to PCL had significant effects on the particle size and *EE* of TPR-NPs. Low ratio of drug could result in smaller particle size of TPR-NPs and higher *EE*. It may be caused by the participation of drug that reduces the emulsifiability of oil phase (Lu et al., 2015). Considering the merits of high drug loading and small particle size for application, 7.5% drug/PCL ratio was decided in the final formulation.

**Figure 1.**
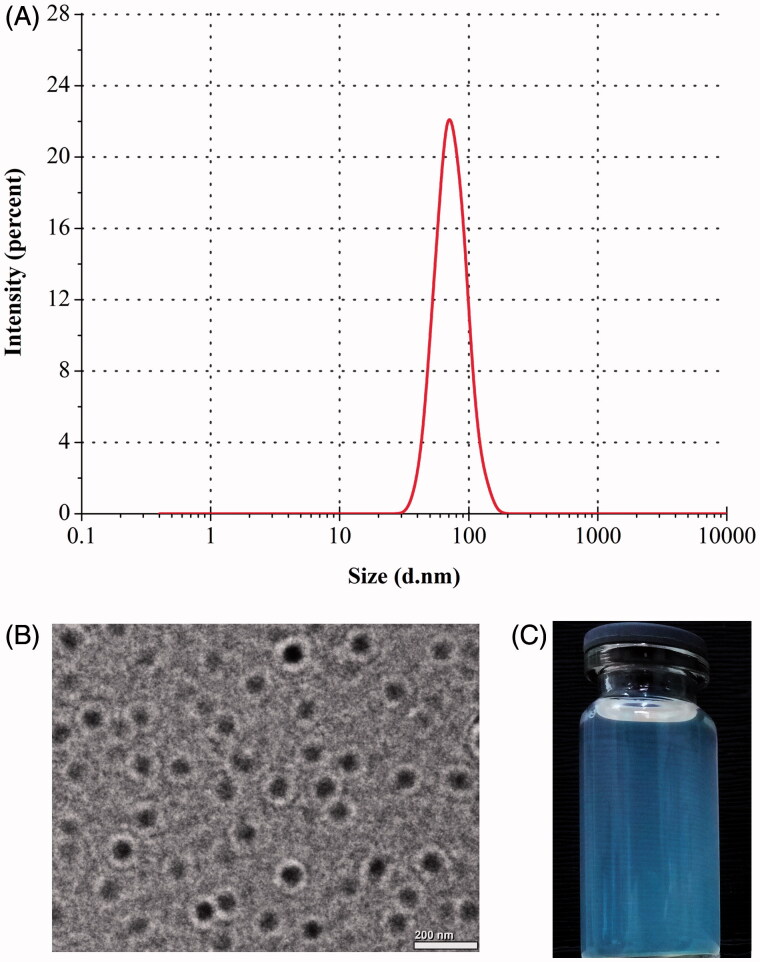
Particle size distribution (A), TEM morphology (B), and appearance (C) of TPR-NPs prepared from the optimal formulation.

TPR-NPs produced using the above formulation displayed a particle size of 75.4 nm with a polydispersity of 0.174 (Figure 1(A)). TPR-NPs were spherical in morphology as revealed by TEM (Figure 1(B)). The particle size estimated by TEM was slightly larger than the hydrodynamic size determined based on DLS principle, probably as a result of longitudinal compression upon dehydration in the process of sample preparation. Nevertheless, the size of TPR-NPs presented by TEM was below 100 nm as gauged by the scale bar. An apparent bluish opalescence was provided with TPR-NPs (Figure 1(C)). By condensing, TPR concentration could reach 2 mg/mL in the system of TPR-NPs with no significant increase of particle size, which can satisfy for the requirements of injection volume and dose.

### 3.2 *In vitro* drug release

**Figure 2.**
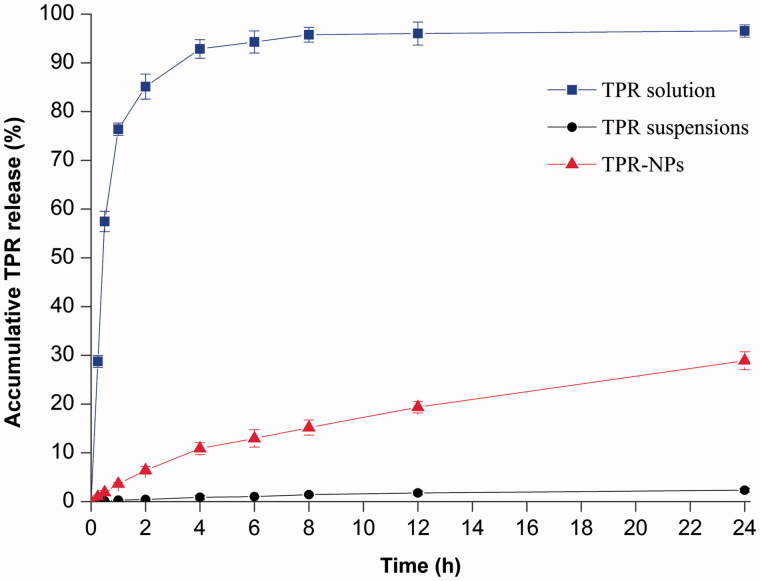
Release profiles of TPR from solution, suspensions and TPR-NPs determined by the reverse bulk equilibrium dialysis method (*n* = 3, mean ± SD).

Figure 2 shows the release profiles of TPR solution, TPR suspensions, and TPR-NPs. In terms of TPR solution, the drug concentration came to balance in a short time on both sides of the dialysis bag, indicating less release resistance provided by the dialysis membrane. However, TPR suspensions, simply dispersed in 5% CMC-Na solution, exhibited an extremely slow drug release. The accumulative release percentage at 24 h was merely 2.32%, which is attributed to high hydrophobicity of TPR (Zhang et al., 2014b). Compared with TPR suspensions, TPR-NPs showed a relatively fast drug release behavior, but significantly slower than that of TPR solution. In pH 7.4 medium, approximately 28% of TPR was released from TPR-NPs at 24 h, and the release accorded with the process of first-order kinetics (*r*^2^ = 0.9913), indicating that TPR can be continuously released from NPs. Although the *in vitro* release determined with the dialysis bag did not fully reflect the actual condition of TPR-NPs release in the blood stream, it was helpful to understand the *in vivo* fate of NPs-associated drug.

### 3.3 *In vitro* antitumor activity

**Figure 3.**
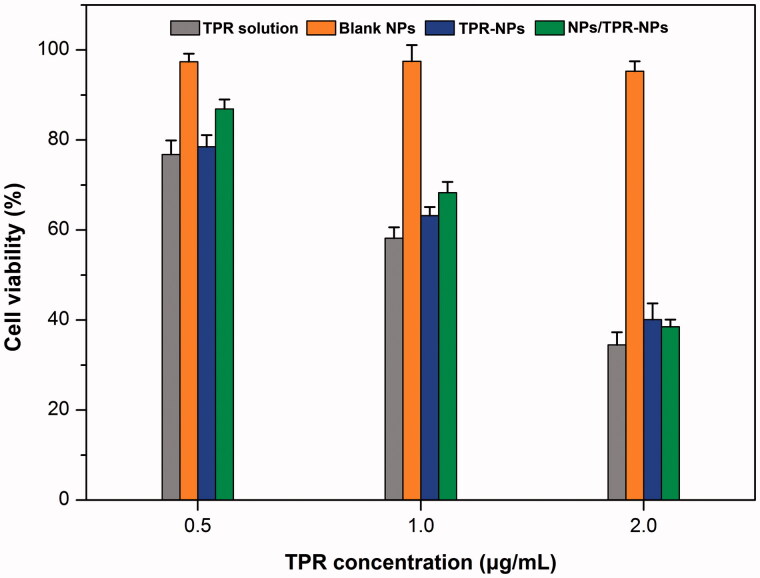
Cell viability of LNCaP cells after treatment with TPR solution, blank NPs, TPR-NPs, and NPs/TPRNPs for 24 h at different concentrations besides blank NPs.

The cell viability of LNCaP cells after treatment with TPR solution, blank NPs, TPR-NPs, and NPs/TPR-NPs for 24 h is presented in Figure 3. Blank NPs almost posed no cytotoxicity on LNCaP cells, and the survival rate maintained above 95%. Free TPR in the form of solution exhibited strong inhibitory effect at three different TPR concentrations, resulting in 76.8%, 58.2%, and 34.5% decline in cell viability, respectively. For TPR-NPs, the inhibitory activity on LNCaP cells was almost parallel to that of TPR solution, which did not exhibit more potent antitumor activity. It is assumed that high lipophilicity enable TPR to be readily transported into the cells and thus cause cell apoptosis. Likewise, TPR-NPs can be internalized into cells and induce cell apoptosis by releasing the payload in the lysosome (Zhang et al., 2017). The cell viability in the group of NPs/TPR-NPs was a little higher than that of TPR-NPs alone at 0.5 h and 1 h. But, the combined use of NPs and TPR-NPs almost exhibited similar cytotoxicity to TPR-NPs at 2 h. This may be associated with non-specific cytosis of NPs and TPR-NPs that causes reduced uptake of TPR-NPs in the initial time. However, the effect of NPs on TPR-NPs uptake was counterbalanced in the late stage. Although the *in vitro* antitumor activity of free TPR, TPR-NPs, and NPs/TPR-NPs is comparable in potency, TPR-NPs possess higher safety and biocompatibility for *in vivo* application.

### 3.4 Improved pharmacokinetics and biodistribution with TPR-NPs

**Figure 4.**
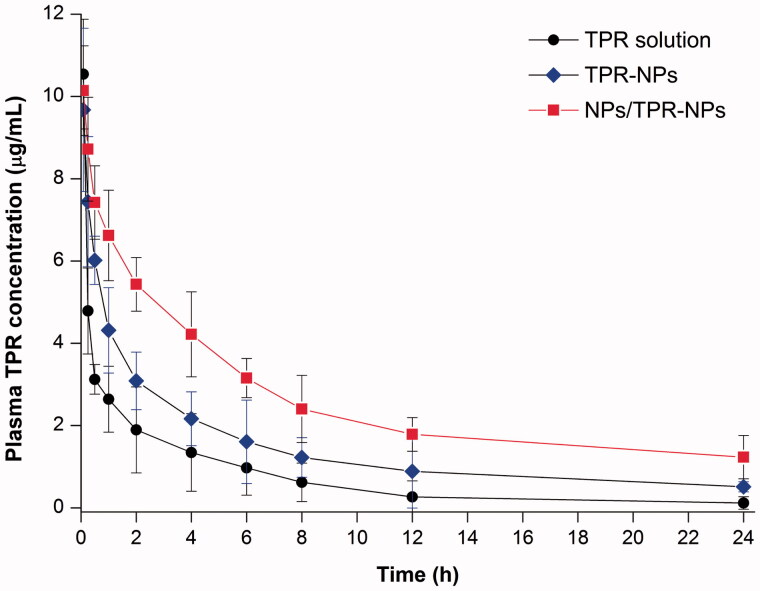
Pharmacokinetic profiles of TPR in rats after intravenous injection of TPR solution, TPR-NPs, and NPs/TPR-NPs (preinjection of blank NPs followed by injection of TPR-NPs). Data expressed as mean ± SD (*n* = 5).

The plasma concentration versus time curves of TPR following intravenous injection of TPR solution, TPR-NPs without or with preinjection of NPs are shown in Figure 4. Free TPR showed a rapid decline in the plasma level. TPR-NPs improved the pharmacokinetics of TPR to some extent with a reduced decline of plasma drug concentration. In the case of TPR-NPs alone, the pharmacokinetic improvement for TPR was not satisfactory relative to the solution formulation. When pretreated with NPs prior to TPR-NPs injection, the plasma concentrations of TPR were significantly elevated in comparison with pure injection of TPR-NPs. The main pharmacokinetic parameters are summarized in Table S1. With preinjection of blank NPs, the area under plasma concentration-time curve (*AUC*_0−t_), total clearance from plasma (*CL*), terminal half-life (*T*_1/2_), and mean residence time (MRT) of TPR were significantly greater than those resulted from TPR-NPs alone. From these results, it can be affirmed that TPR-NPs have the potential to improve the pharmacokinetic profile of TPR, whereas preinjection of blank NPs can further the improvement.

**Figure 5.**
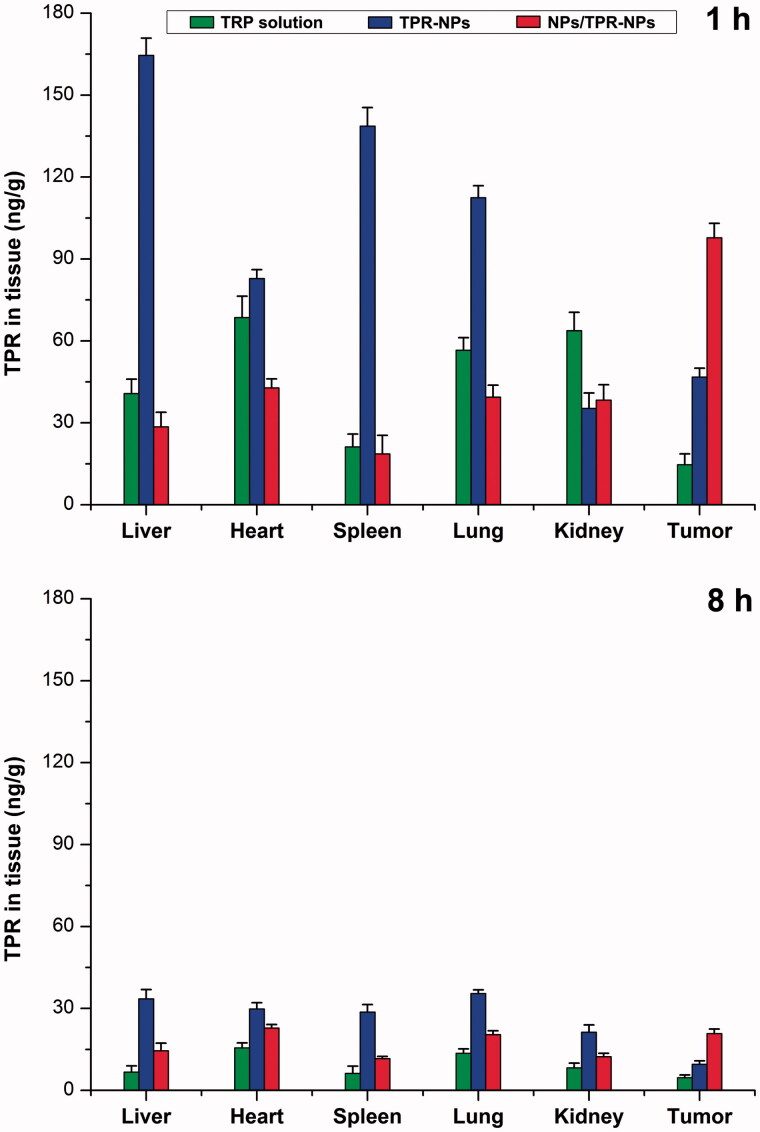
Tissue distribution of TPR in tumor-bearing mice at 1 and 8 h after injection of TPR solution, TPRNPs or NPs/TPR-NPs. Data shown as mean ± SD (*n* = 5).

Improved pharmacokinetics via RES saturation can also be verified by the biodistribution performed in tumor-bearing mice. Figure 5 presents the tissue distribution of TPR at 1 and 8 h after administration of TPR solution, TPR-NPs, and TPR-NPs with preinjection of blank NPs. Apparently, relative to free TPR, TPR-NPs resulted in high distribution into the liver, spleen and lung featured by MPS (Lucas et al., 2017). It is because that NPs are more easily sequestrated by the monocytes and macrophages than small molecules. Reversely, NPs tend to accumulate toward the tumor tissue due to the EPR effect. Accordingly, TPR-NPs exhibited high drug level in the tumor compared with free TPR. However, higher distribution in the tumor site took place on the group of NPs/TPR-NPs that had a preinjection of blank NPs. The cause lies in the fact that preinjected NPs can first fill up the RES and thereby make TPR-NPs less being captured by phagocytes. Inverse targeting, a means of RES saturation, was originally discovered and proposed by Gamble’s group (Proffitt et al., 1983). It referred to a reversible RES blocking whereby to detain the later injected drug-loaded NPs in the circulatory system through injection of blank NPs early, thus increasing accumulation of drug-loaded NPs toward non-RES tissues. At 8 h after injection, TPR in various tissues significantly declined relative to 1 h. However, NPs/TPR-NPs still remained a higher TPR level in the tumor tissue than free TPR and TPR-NPs. The findings of optimized pharmacokinetics and biodistribution confirm the feasibility of RES saturation for passively delivering anticancer agents to the tumor site.

### 3.5 Enhanced antitumor effect of TPR-NPs via RES saturation

**Figure 6.**
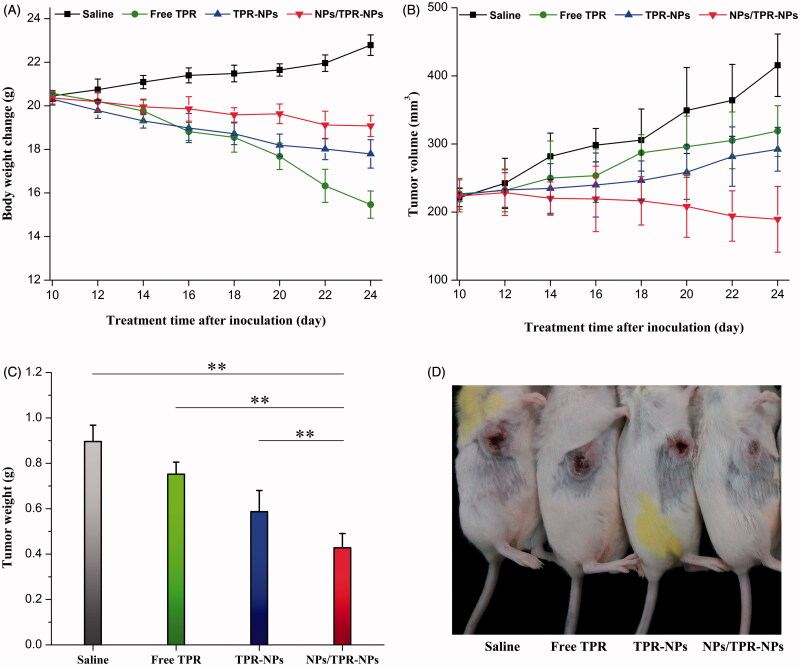
*In vivo* antitumor effects of free TPR, TPR-NPs, and NPs/TPR-NPs in LNCaP tumor xenograft mice: (A) body weight changes of mice with the treatment time; (B) tumor volume changes of mice with the treatment time; (C) average tumor weight at the end of experiment (ANOVA, **p < .01, compared with saline, free TPR, TPR-NPs, and NPs/TPR-NPs); (D) Typical mice with developed tumor after treatment for 14 d.

The body weight, tumor volume, average tumor weight, and representative mice with tumor progression after treatment for 14 d are shown in Figure 6. The body weight of mice treated with saline progressively increased (Figure 6(A)), which may be connected with the rapid growth of tumor. In sharp contrast, the mice receiving TPR solution injection suffered from a great body weight loss. It was possibly caused by the high toxicity of TPR itself and the involvement of harmful solvents used to dissolve TPR. Compared with free TPR, the body weight loss of mice was suppressed by TPR-NPs, indicating that NPs can attenuate the systemic toxicity of TPR. However, the body weight declined less in the case of NPs/TPR-NPs relative to TPR-NPs alone. This implied that the tumor growth of mice was inhibited due to combined treatment of NPs/TPR-NPs. Figure 6(B) shows the tumor volume changes with the treatment time. The tumor volume of mice grew large gradually in the groups of saline, free TPR and TPR-NPs. Conversely, the tumor volume of mice treated with NPs/TPR-NPs started to decline after the second injection of NPs/TPR-NPs. The antitumor effect of NPs/TPR-NPs had the overwhelming advantage over TPR-NPs alone. From the outcomes of tumor weight after treatment (Figure 6(C)), it could be inferred that the preinjection of NPs played a positive role in enhancing the curative effect of TPR-NPs. Figure 6(D) shows the *in situ* tumor changes in mice after treatment, which also demonstrates that NPs/TPR-NPs possess stronger tumor growth inhibition. Enhanced antitumor efficacy of NPs/TPR-NPs can be attributed to optimized distribution of NPs through preinjection of blank NPs that reduces uptake of later injected TPR-NPs by the RES and smaller particle size that reduces identification by mononuclear macrophages, thus increasing the passive targeting of TPR-NPs via the EPR effect (Huynh et al., 2011).

To further understand the progression of tumor development, H.E. staining was performed to check the histomorphology of tumor after treatment of 14 d. Figure S3 displays the typical pathological histology of tumor sections from the mice treated with saline and NPs/TPR-NPs. Obviously, control mice exhibited a normal tumor cell morphology and intact nucleus. However, the tumor from NPs/TPR-NPs group took on a distinct cell metamorphosis. Characteristic cell swelling, vacuolar degeneration, nucleus contraction, and agglomeration were observable. It is indicative that the tumor cells have become apoptotic, and the tumor shows signs of necrosis. To circumvent the unfavorable properties of TPR, Sanna et al. (2015) formulated into polymeric NPs by nanoencapsulation used for cancer treatment, finding TPR-NPs more suitable for prostatic cancer treatment. TPR-loaded polymeric micelles have also been reported able to inhibit the growth of retinoblastoma in a xenograft model by inducing apoptosis of SO-Rb 50 cells (Li et al., 2012). The *in vivo* antitumor study demonstrates the suitability of RES saturation or reverse targeting for systemic delivery of TPR on the base of NPs.

## 4. Conclusions

In this work, polymeric NPs were developed for systemic delivery of TPR in combination with preinjection of blank NPs. TPR-NPs with smaller size and high *EE* were successfully prepared by the low-energy emulsification/evaporation method. TPR-NPs possessed a favorable drug release character compared with the solution formulation. The *in vitro* antitumor activity of TPR has not been impaired even if encapsulated into NPs. RES saturation could effectively improve the pharmacokinetics and biodistribution of TPR by prolonging the systemic circulation of TPR-NPs. Significantly enhanced antitumor effect of TPR was achieved by preinjection of blank NPs followed by injection of TPR-NPs. This study provides new insight into the use of polymeric NPs for prostate cancer treatment based on the physiological principle.

## Supplementary Material

IDRD_Yin_et_al_Supplemental_Content.pdf
