# Supplementary material for: Optimization on biodistribution and antitumor activity of tripterine using polymeric nanoparticles through RES saturation
Source: Drug Deliv. 2017 Dec 1;24(1):1891–7. doi: 10.1080/10717544.2017.1410260 (PMC8241100; doi:10.1080/10717544.2017.1410260)
Supplement: IDRD_Yin_et_al_Supplemental_Content.pdf [file IDRD_A_1410260_SM8068.pdf]

Supporting information for:

Optimization on biodistribution and antitumor activity of tripterine using polymeric nanoparticles through RES saturation

Juntao Yin [a], Peiqing Wang [a], Yuyun Yin [b], Yantao Hou [c], Xiaoyong Song [a,*]

[a] Department of Pharmaceutics, Huaihe Hospital Affiliated to Henan University, No. 1 Baobei Road, Kaifeng 475000, P.R. China.

[b] Henan Provincial Institute of Food and Drug Control, No. 8 Jinger Road, Zhengzhou 450003, P.R. China.

[c] Henan Vocational College of Applied Technology, Dongjing Avenue, Kaifeng 475004 , P.R. China.

***Corresponding author:** Department of Pharmaceutics, Huaihe Hospital Affiliated to Henan University, No. 1 Baobei Road, Kaifeng 475000, P.R. China.

Tel.: +86 0378 23906304

E-mail: sxyyjk@sina.com

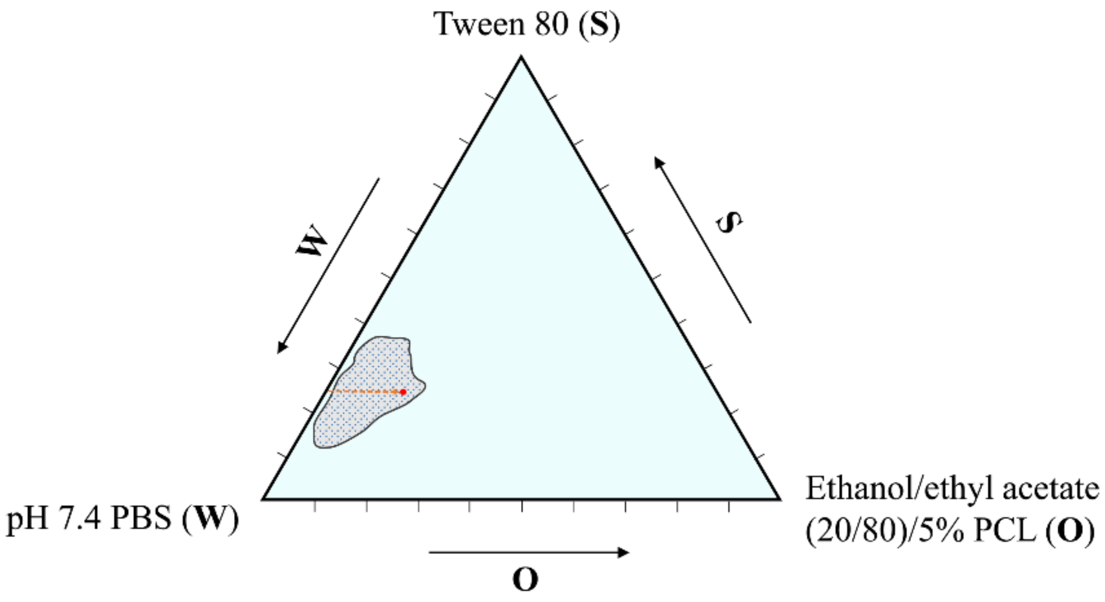

Figure S1. Pseudoternary phase diagram of oil, water and surfactant showing O/W nanoemulsions region.

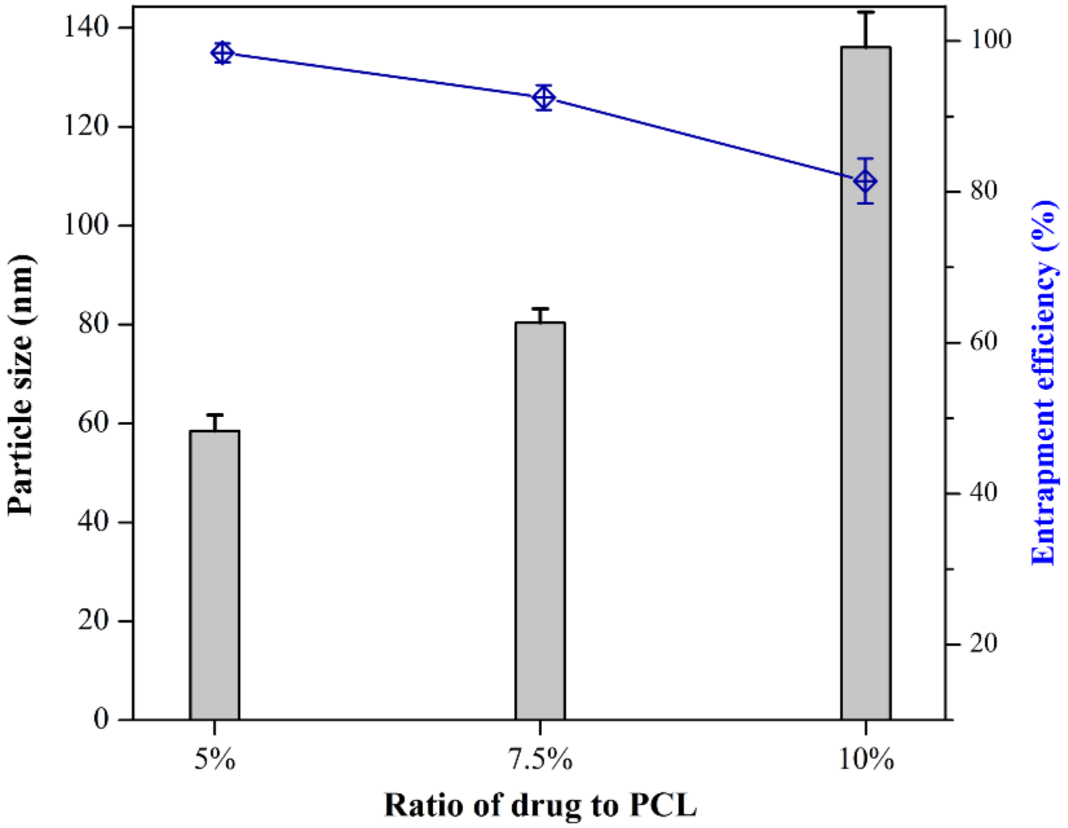

Figure S2. Effect of TPR/PCL ratio on the particle size and entrapment efficiency of TPR-NPs (n = 3, mean ± SD).

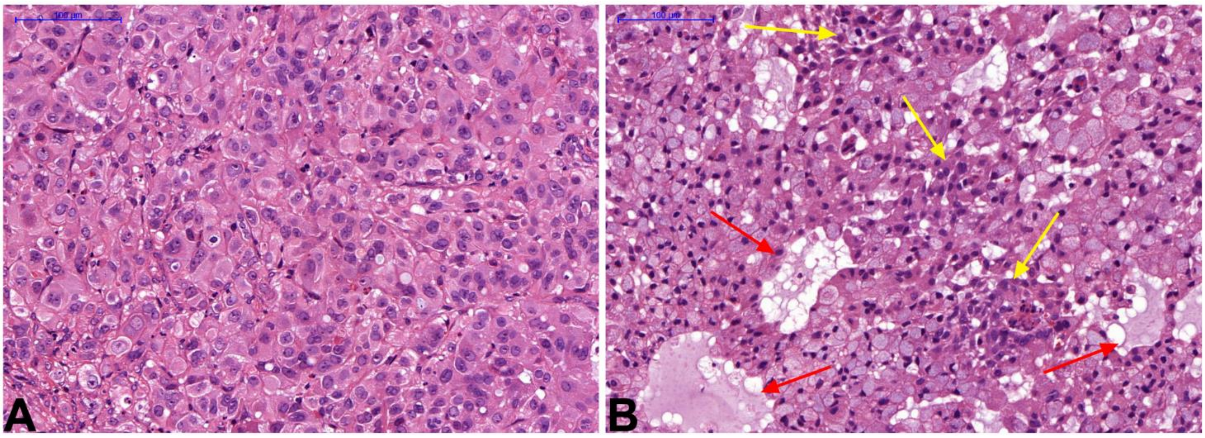

Figure S3: Histomorphological features of tumor masses after treatment: control group presenting a normal state, whereas NPs/TPR-NPs group showing significant cell swelling, vacuolar degeneration (red arrow), nucleus contraction and agglomeration (yellow arrow).

Table S1. Pharmacokinetic parameters of TPR in rats after intravenous injection of TPR solution, TPR-NPs and NPs/TPR-NPs.

Parameter	Free TPR	TPR-NPs	NPs/TPR-NPs
**$AUC_{0\text{-}t}$ (μg·h/mL)**	4.532 ± 0.321	18.45 ± 0.477	49.23 ± 0.425**
***CL* (L/h)**	0.552 ± 0.164	0.135 ± 0.089	0.050 ± 0.002**
***Vd* (L)**	0.204 ± 0.08	0.291 ± 0.135	0.283 ± 0.068
**$T_{1/2}$ (h)**	0.257 ± 0.102	1.491 ± 0.310	3.919 ± 0.826**
***MRT* (h)**	0.371 ± 0.174	2.150 ± 0.343	5.654 ± 0.482**

Note: NPs/TPR-NPs denoting preinjection of blank NPs followed by injection of TPR-NPs after 30 min. Data expressed as mean ± SD (n = 5). Statistical analysis: paired-t test, ** P < 0.01, significantly different from TPR-NPs.
